# Medico-Chirurgical Transactions

**Published:** 1816-08

**Authors:** 


### *Medico-Chirurgical Transactions, Vol vi.*

( Continued from page 40. )

### *Further Observations on the Ulceration of the Cartilages of the Joints.* By Mr. Brodie.

This is a long paper, and full, as usual, of minute, though doubtless, useful distinctions between the different affections of the joints. Few, however, will retain any part of them in their memory.

Ulceration of the cartilages, Mr B. remarks, is sometimes primary, sometimes secondary. It is the former to which this paper is directed. It takes place at any period of life, but principally in children, or under middle age. The Hip is the joint most prone to this affection. It may sometimes be traced to local injury or cold, but generally no cause can be assigned. The first symptoms (in the hip cases) are pain and slight lameness in the lower limb, resembling rheumatism, from which it is difficult to distinguish it. As the disease advances, the pain becomes exceedingly severe, especially at night, and is sometimes relieved by a particular position. It is now more circumscribed in site, and better defined. It is aggravated by motion; there is tenderness on pressure, and, as the disease becomes still more advanced, there is a fulness in the groin. Sympathetic pains in different parts are equally common and unaccountable. The nates now become altered in appearance; they are wasted, flattened, and flaccid. This arises from wasting of the glutei.

Alteration in the length of the limb is another symptom. 1st. In advanced stages, where there is extensive destruction of the head of the femur, and bony margin of the acetabulum, the limb is shortened. 2d. In some cases, where the joint is filled with coagulable lymph, without much destruction of the bones, the head of the femur is pushed outwards till it gets beyond the margin of the acetabulum, and becomes in fact *dislocated upwards and outwards.** The limb is here shortened; the thigh bent forwards; the toes turned inwards, as where there is a common dislocation. It is generally succeeded, sooner or later, by abscess in the joint.

In the early stage of the disease, there is sometimes an apparent elongation of the limb, produced by the position of the pelvis being altered, so as no longer to make a right angle with the spine. In other cases, there is an *apparent* shortening of the limb, from the same cause. It is of great importance to distinguish this *apparent* from that real shortening of the limb in advanced stages of the disease indicating abscess. The health usually suffers anterior to abscess, from the pain, want of exercise and of rest. After abscess, hectic fever closes the scene; except in a few infantile cases, where anchylosis rescues the patient from the grave.

Where the cartilages of the knee are ulcerated, the pain is confined to the affected joint; at first slight, and relieved by rest; ultimately constant, and very severe at night. The pain is principally referred to the inside of the head of the tibia; is aggravated by motion, but unattended by rigidity, except in an advanced stage.

*This* differs from inflammation of the synovial membranes, since in it (ulceration of cartilages) the pain is at

* If this succeed a trifling accident in a child, may not the surgeon of the family be innocently accused of neglecting a dislocation, and the whole blame be thrown on him by another practitioner, consulted at a later period? Editors.

first slight, and ultimately intense; the reverse of what takes place in synovial inflammation. Ulceration also is unattended with swelling for some weeks, or even months, after the commencement of the disease, unlike most other affections of the knee-joint. The progress of this disease is generally tedious.

TREATMENT. Rest is the most important circumstance to be attended to in the treatment. Caustic issues, and blisters kept open by savine cerate, &c. are useful; so are setons. In the early stage, the warm bath is beneficial. Friction is injurious.

*Ulceration of the Cartilages of the Hip anterior to Suppuration.*

The patient to be confined to his couch or bed; the limb properly supported by pillows, so as to favour the production of anchylosis. Blisters on the nates; round the great trochanter, and in the groin, kept *constantly* open. In the advanced stages, caustic issues are preferable, in the hollow behind the great trochanter. Mr. Brodie has found the sore best kept open by touching its surface, twice or thrice a week, with sulphate of copper, and not using beans at all. If there be violent pain, local bleeding may be necessary; and a blister to the knee will often relieve, *from sympathy*, the pain in the hip. This is Mr. Brodie's opinion: we think it is by producing a *determination* to another part.

A seton in the *groin* is very useful, probably from being so near the joint. The same remarks apply to the disease on the knee or any other joint, making allowance for difference of site.

In respect to the disease, after the period of suppuration, it is hopeless in adults; but not so in children. The method of Abernethy, in lumbar abscess, has not been of any use in these cases. The *early* puncture of articular abscess is not advisable. Quietude, and the means above recommended, should be insisted on, previously to the opening of an abscess in a joint. These abscesses, particularly in the hip-joint, do not form regular cavities, but make circuitous sinuses in the interstices of the muscles, tendons, and fasciæ, before they present under the integuments; the matter, therefore, cannot be evacuated without such pressure and handling of the joint as frequently bring on inflammation and great irritation. The best practice is, to open the abscess with a lancet; to wrap up the limb in flannel wrung out of hot water, and to continue this fomentation as long as the matter flows, leaving the orifice to heal of itself, or not.

We have thus extracted the pith or essence of Mr. Brodie's paper. We have said that the distinctions pointed out *will* not be long remembered by practitioners in general; but we are quite sure that they *ought* to be attended to.

*Case of Hernia Ventriculi from external Violence, wherein the Diaphragm was lacerated without Fracture of the Ribs.* By THOMAS WHEELWRIGHT, Esq.

GERARD GROUT, a stout young seaman, was thrown from the top of a coach between Plymouth and London, but did not complain of much injury, and continued his journey to town. On the evening of the next day, he was taken very ill, with pain in the left side, sickness, and dyspnœa. On examination, no fracture of a rib or other bone could be perceived. Pulse being strong, a pound of blood was abstracted. At ten o'clock of the third day from the accident, Mr W. saw him. He then suffered severely from the same symptoms as above, with the addition of bloody vomiting. Pulse 120, and small; skin cold. He expired the same evening.

*Sectio Cadaveris.* No extravasated blood in the abdomen. The pyloric orifice of the stomach somewhat inflamed, and unusually confined. In the thorax, three pints of blood in the left side; a considerable portion of the large curvature of the stomach protruded through a fissure of the diaphragm, filled with a sort of half coagulated blood. A small semicircular aperture was found in the strangulated portion of the stomach, through which the blood had slowly escaped into the cavity of the chest. —We have condensed this article, though it tends to no practical or speculative purpose, but merely is a matter of curiosity. If worthy of seven pages in the Medico-Chirurgical Transactions, it cannot be considered an intrusion to give it half a page here.

*Sketch of the Medical History of the British Armies in the Peninsula of Spain and Portugal during the late Campaigns.* By Sir JAMES M'GRIGOR, M. D. &c.

ALTHOUGH this article occupies one hundred pages of the volume before us, we hesitated about the propriety of offering to our readers any analysis either of it or the succeeding paper by Sir Gilbert Blane: yet, as some degree of the *milito-mania* may still lurk in the bosoms of our younger medical brethren, we shall take a rapid survey of one part of this communication, namely, that which contains Sir James's "Remarks upon the Diseases which prevailed," leaving the history and military hygiene of the campaigns for the perusal of those whose former avocations, or future prospects, may render them interesting. To us, and to the great bulk of our readers, retired in the private walks of life, and occupied with the duties of our profession, these tumultuous scenes can offer nothing but the gratification of curiosity.

*Fever*. There is little satisfactory to be gleaned from this head. Dr. M'Grigor informs us, as indeed we all know, that fever differs in different situations, seasons, and other circumstances, requiring different, and even directly opposite modes of treatment. Intermittents were very common, and, when early taken, yielded to bark, arsenic, or sulph. zinci, which were powerful in proportion as they here stand. Sulph. zinci was given in the form of pills, and often to the amount of half a drachm a day. An ounce of bark, a table spoonful of Jamaica pepper, and a whole nutmeg, given in one dose, often succeeded, when other means failed. Where visceral disease existed, mercury of course was necessary. Dr. M'Grigor accuses mercury, "improperly and nearly indiscriminately used," of causing subsequent debility after this disease, and dysentery; "though," says he, "the cautious use of this remedy will, in both these diseases, effect a cure or give relief, when no other medicine will." Dr. M'G. subscribes to an opinion, which is now gaining ground, that "intermittents of long standing, as well as dysenteries, appear to have some connexion with the liver or the biliary secretion." We believe that this connexion exists in dysentery from the beginning, since, in the many thousand cases of dysentery which we have seen, the biliary secretion never appeared natural in any individual, or in any stage of the disease. Some documents, which have lately come before the public respecting *large doses* of calomel in dysentery, will form a pretty effectual set-off against the oblique attack conveyed in the paragraph above, and in various places afterwards.

*Dysentery*. Dr. M'G. here, as in his Medical Sketches, asserts, that there are *two species* of dysentery, tropical and hyperborean. This division of species, according to chart or thermometer, will not now be acknowledged. The day is past in which such doctrines would receive any attention. It was of importance to distinguish two stages, however, of the peninsular dysentery; that of inflammation and of ulceration. In those cases where no pyrexia was visible, our author considered dysentery to be "symptomatic either of disease of the biliary system, or of the mesenteric glands." We believe that no case of dysentery ever yet occurred without more or less of pyrexia at its commencement. "In most cases," says the Doctor, "I think dysentery was accompanied by fever of the inflammatory type." In this we are of the same opinion. We also agree with him, that the type of this fever is modified by the prevailing epidemic. The army in less than three years lost 4717 men by dysentery alone; yet, "when the disease was pure and uncombined, and when the cases were taken early, it was found very manageable in treatment."

The best practice, in primary attacks, was, to commence with copious venæsection, and immediately afterwards to give twelve grains of the pulv. ipecac. comp. every hour, for three or four hours, encouraging perspiration by warm diluting drinks. Three grains of the submur. hydrarg. and one of opium, were given every second night, and on the intervening day ℥ij of the sulphate of magnesia dissolved in a quart of broth. The venæsection was repeated when the strength permitted, till the stools became bloodless, following up this plan with the Dover's powder as a sudorific. Where great pain and tenesmus were complained of, the warm bath gave instantaneous relief. In a few days the disease gave way, when light tonics, and nourishment sparingly exhibited, completed the restoration of health. Relapses were very frequent.

The form in which our author most frequently saw dysentery was, where visceral disease (principally of the liver and spleen, sometimes the mesenteric glands) accompanied it; and here mercury, of course, was the only remedy to be relied on.

"Ipecacuanha, opium, and other articles, were frequently given with calomel, in small and frequently repeated doses; and some gentlemen gave the blue pill until the mouth was affected; but the general opinion was, that friction of the abdomen with mercurial ointment gave least irritation, and at the same time produced less debility."

The various *adjuvantia*, recommended by different authors, were used here to mitigate symptoms; of which the warm bath, blisters, and bandages, appeared most beneficial. Mr. Woolrich, in the hospitals at Celerico, reports favourably of bals. copaibæ and gum Arabic, using occasionally gentle mercurial friction, flannel bandages, &c.

"A great many of the bodies of the dysenterics were examined, and *a great uniformity* was found in the morbid appearances: ulcuscula, which had a healed appearance; purulent collections, formed in various parts of the canal; the liver, in many cases, hardened, of a dark complexion, enlarged, and with preternatural adhesions. In some cases, it appeared smaller than usual; and in these the spleen was large and diseased. In a majority of cases, it was found that the colon, from the arch downwards, and the rectum more especially, were throughout in an ulcerated sloughy state."

Upon the whole, there is nothing new in this paper, respecting the pathology or treatment of dysentery: every remedy here recommended has been prescribed by others (with the exception of bals. copaibæ), and every symptom clearly accounted for, and the modus operandi of the different remedies explained, before Dr. M'Grigor's paper appeared.

*Pneumonia.* The following passage is applicable to more than *military* practice, and we recommend it to all the *old women* of the profession as orthodox doctrine even in *private* practice.

"The military practitioner, in repeatedly extracting blood, is not to be guided by quantity, or even the appearance of the blood, but by the relief procured. Neither blistering, sudorifics, purging, or other remedy, can supply the place of bleeding; nor must he judge by what he has seen in *private practice*, where this sudden abstraction of a large quantity of blood would *perhaps* be improper."

We know not why there should be any *impropriety* in abstracting blood from a *private* patient affected with pneumonia till "relief was procured," regardless of the quantity or appearance of the blood; and we challenge Dr. M'Grigor, or any other man, to offer any good reason for the difference. If in *civil* life the disease does not generally run so high as in *military*, nor require such copious abstractions of blood *before relief is procured*, yet the principle of practice is not altered in the least.

An obscure, but dangerous form of this disease, which "does not shew itself in its natural colours till the functions of the oppressed and congested lungs are, in some degree, restored by abstraction of blood," is not, as our author imagines, "nearly peculiar to soldiers." Those whose duties, inclinations, or humanity, lead them through the haunts of poverty and sickness in this capital, or in any of the larger towns of Great Britain, will meet the above-mentioned disease, and others of a similar nature; but where the brain, liver, or some important organ, is in the same state as the lungs are represented to be in by Dr. M'Grigor—it is in these forms of *masked debility* that the *routinist* displays the destructive power of medicine in all its terrors; and woe to the wretched patient who has the misfortune to swallow his camphor mixtures, bark, opium, and æther!

*Phthisis.* Some very interesting remarks occur under this head. Sir James's evidence is highly inimical to the doctrine of Dr. Sutton, and affords strong support to the opinions of Dr. Powell. Only 279 deaths from phthisis occurred in three years throughout the whole of the general and regimental hospitals of the peninsular army—

"A small proportion, and very widely different from what I have found to be the case in the army in England. Under ordinary circumstances, and when no epidemic or contagious disease prevailed, I found in England the deaths from consumption amount to one-fifth, one-fourth, and, in some regiments, even as high as one-half of the whole mortality."

Surely this is conclusive evidence, that consumption originates more frequently in England than in the warmer countries of Europe, or between the tropics. In respect to phthisis being less prevalent where intermittents prevailed, the following was the result of our author's inquiries:

"In the period included from the 25th of June to the 24th of September, 1812, it appears that the cases of phthisis, at six hospital stations, amount to 54; and of remittent and intermittent fever, 1880; therefore the proportion of phthisis pulmonalis to remittent and intermittent fever is as 1 to 35." p. 441.

And again,

"It clearly appears, that in the early stage of consumption, that is to say, when suppuration and ulceration have not yet taken place, the disease is checked by the climate of the peninsula; but that when suppuration and ulceration have taken place, it runs even a more rapid progress than in England; and I have made the same remark in regard to the East and West Indies." p. 442.

With these observations we entirely coincide; and every practitioner may trace analogies to them without going from home. Do we not see phthisical patients *anterior to purulent expectoration* rally every summer, and fall back in the winter and spring; but *after* ulceration, we see the heat of summer so much overcome them, that they generally drop off in that season. Is there any thing unaccountable in this? Assuredly not. The result of Sir James's experience in Walcheren is corroborative of Dr. Powell's hypothesis.

*Rheumatism.* The only novelty under this class of disease is, the practice of Mr. Paddock at Santarem, who, in old chronic cases, which had resisted the usual treatment, administered ol. terebinth. ʒj bis die. He occasionally gave half an ounce as a purge.

*Tetanus.* This formidable disease made dreadful havoc in our peninsular army. Among several hundred cases, very few seemed at all influenced by medicine, where the disease had made any progress. There is a remarkable instance, where unavoidable exposure to wet and cold apparently saved the patient. It was severe tetanus, from a wound in one of the fingers. The man was carried after the battalion in a bullock car; and, during the first part of the day, was drenched with rain, the thermometer standing at 52°: but after ascending the highest mountains in Gallicia, the snow was knee-deep, and the mercury below 30. The patient was exposed to this inclement weather from six in the morning till ten at night, when he arrived half dead with cold, but perfectly free from every symptom of tetanus!

In another successful case, the treatment was twenty drops of laudanum every second hour; and in the intermediate hour, a dose of carbonate of potash, not exceeding fifteen grains, in mint water. He recovered perfectly in about three weeks.

One of the German corps was treated in nearly a similar manner, and was saved. They were both traumatic tetanus. At Elvas, after the battle of Albuera, "opium was the *favourite* remedy, *and every case so treated died.*" This had certainly no great title to the epithet "*favourite.*"

Two stout young officers, after wounds, "were attacked with violent spasms about the throat, and the jaw began to be affected in both." Emollient poultices to the wounds, bleeding, purging, and the antiphlogistic regimen strictly observed, removed these alarming symptoms. Col. Sir George Elder was successfully treated by opium, calomel, mercurial friction to ptyalism; tinct. opii rubbed over the body in large quantities; wine, and spirits in the form of punch, liberally given; and daily purgatives.

The evacuations were always copious, dark coloured, and highly offensive. It was observed by the medical officers of the army, that twenty-two days' immunity from tetanus, after the date of a wound, was a safeguard from an attack.

Venæsectio ad deliquium appeared in a few instances to save the patient from this terrible disorder, but in many others it totally failed. Mercurial purgatives, opium, and digitalis, may rank in success as they here stand.

"I am, however, obliged to confess, that little or no dependence is to be placed in any of the remedies; and I have to regret that the method of cure is yet to be discovered."

The third part of our author's paper, on Military Hygiène, though interesting and important to military readers, is not so to the private practitioner; and therefore we shall pass it over. On the whole, this article detracts nothing from the high, and deservedly high, professional reputation of Sir James M'Grigor.

(To be continued.)

